# Down-Regulation of Tinnitus Negative Valence via Concurrent HD-tDCS and PEI Technique: A Pilot Study

**DOI:** 10.3390/brainsci13050826

**Published:** 2023-05-19

**Authors:** Zahra Vaziri, Carlos E. G. Salmon, Iman Ghodratitoostani, Antonio Carlos dos Santos, Miguel A. Hyppolito, Alexandre C. B. Delbem, João P. Leite

**Affiliations:** 1 Department of Neuroscience and Behavior, Faculty of Medicine of Ribeirão Preto, University of São Paulo, Ribeirão Preto 14048-900, Brazil; jpleite@fmrp.usp.br; 2 InBrain Lab, Department of Physics, Faculty of Philosophy, Sciences and Letters, University of São Paulo, Ribeirão Preto 14040-901, Brazil; garrido@ffclrp.usp.br; 3 Neurocognitive Engineering Laboratory, Center for Engineering Applied to Health, Institute of Mathematics and Computer Science, University of São Paulo, São Carlos 13566-590, Brazil; iman.ghodrati@alumni.usp.br (I.G.); acbd@icmc.usp.br (A.C.B.D.); 4 Department of Medical Imaging, Hematology and Clinical Oncology, Faculty of Medicine of Ribeirão Preto, University of São Paulo, Ribeirão Preto 14049-900, Brazil; acsantos@fmrp.usp.br; 5 Department of Ophthalmology, Otorhinolaryngology, Head and Neck Surgery, Faculty of Medicine of Ribeirão Preto, University of São Paulo, Ribeirão Preto 14049-900, Brazil; mahyppo@fmrp.usp.br

**Keywords:** high-definition transcranial direct current stimulation, positive emotion induction, functional magnetic resonance imaging, neurofunctional tinnitus model, dorsolateral prefrontal cortex, tinnitus handicap inventory

## Abstract

Around 30% of the general population experience subjective tinnitus, characterized by conscious attended awareness perception of sound without an external source. Clinical distress tinnitus is more than just experiencing a phantom sound, as it can be highly disruptive and debilitating, leading those affected to seek clinical help. Effective tinnitus treatments are crucial for psychological well-being, but our limited understanding of the underlying neural mechanisms and a lack of a universal cure necessitate further treatment development. In light of the neurofunctional tinnitus model predictions and transcranial electrical stimulation, we conducted an open-label, single-arm, pilot study that utilized high-definition transcranial direct current stimulation (HD-tDCS) concurrent with positive emotion induction (PEI) techniques for ten consecutive sessions to down-regulate tinnitus negative valence in patients with clinical distress tinnitus. We acquired resting-state functional magnetic resonance imaging scans of 12 tinnitus patients (7 females, mean age = 51.25 ± 12.90 years) before and after the intervention to examine resting-state functional connectivity (rsFC) alterations in specific seed regions. The results showed reduced rsFC at post-intervention between the attention and emotion processing regions as follows: (1) bilateral amygdala and left superior parietal lobule (SPL), (2) left amygdala and right SPL, (3) bilateral dorsolateral prefrontal cortex (dlPFC) and bilateral pregenual anterior cingulate cortex (pgACC), and (4) left dlPFC and bilateral pgACC (FWE corrected *p* < 0.05). Furthermore, the post-intervention tinnitus handicap inventory scores were significantly lower than the pre-intervention scores (*p* < 0.05). We concluded that concurrent HD-tDCS and PEI might be effective in reducing tinnitus negative valence, thus alleviating tinnitus distress.

## 1. Introduction

Tinnitus is conscious attended awareness perception (CAAP) of the sound in the absence of an external source [1]. About 30% of the global population experience tinnitus with a subgroup of 3–6% experiencing tinnitus distress [2,3]. To explain why some people experience tinnitus as distressing, various theoretical models have been developed, including cognitive and behavioral models [1,4,5,6].

Hallam, McKenna [7] suggested that negative appraisal and emotional significance of the signal cause heightened arousal leading to failure in habituation. Thereafter, the aversive emotional state of tinnitus was rationalized based on classical conditioning [4]. Subsequently, Zenner, Pfister [5] then highlighted that tinnitus sensitization arises from the interpretation of the sound as unpleasant, fear-inducing, and unpredictable, leading to maladaptive coping and helplessness [5,8]. Furthermore, cognitive misunderstanding of tinnitus has been shown in McKenna, Handscomb [6]‘s study to cause distress and physiological arousal, which result in a distorted perception of sensory input [6].

More recently, Ghodratitoostani, Zana [1] proposed the neurofunctional tinnitus model (NfTM), categorizing tinnitus patients into neutral and clinical distress stages. The NfTM suggests that the evaluative conditional learning (ECL) mechanism plays a role in developing tinnitus-related valence, where neutral tinnitus paired with negative stimuli obtain a negative valence, causing distress Ghodratitoostani, Zana [1]. On the other hand, the NfTM proposes that the CAAP of tinnitus accompanied by positive emotion induction (PEI) might lower negative valence and resulting distress Ghodratitoostani, Zana [1]. The prefrontal cortex is responsible for continuously evaluating tinnitus valence, comparing it with other sensory and auditory inputs, and monitoring persistent perception. The left hemisphere is believed to prevail over positive emotions, while the right hemisphere dominates negative ones.

The neurofunctional tinnitus model (NfTM) suggests that the prefrontal cortex continuously assesses the emotional value of tinnitus and compares it to other sensory inputs Ghodratitoostani, Zana [1], with the dorsolateral prefrontal cortex (dlPFC) being associated with cognitive–emotional valuation, particularly during the down-regulation of negative emotional conditions [9]. Additionally, the brain asymmetry model proposes that the left hemisphere processes positive emotions and the right hemisphere processes negative emotions [10,11].

Furthermore, electroencephalography (EEG) and functional magnetic resonance imaging (fMRI) studies have illustrated that high levels of baseline activity in the left prefrontal cortex (PFC) brightened the prospects of suppressing negative emotions [12,13,14,15]. The down-regulation of negative emotional processing by transcranial direct current stimulation (tDCS) has been reported following anodal stimulation in some studies [16,17,18].

tDCS has been proposed as a potential treatment for tinnitus, with multiple studies investigating its effectiveness. Vanneste, Plazier [19] reported that tDCS led to a significant reduction in tinnitus severity and improved quality of life. Vanneste and De Ridder [20] found that patients who responded to bifrontal tDCS had higher baseline functional connectivity strength. Vanneste and De Ridder [20] noticed that bifrontal tDCS was more effective than EEG-driven tDCS for tinnitus treatment. Teismann, Wollbrink [21] observed that the combination of tDCS and tailor-made notched music training led to a significant reduction in tinnitus-related distress. Yadollahpour, Mayo [22] showed that a chronic protocol of bilateral tDCS over the auditory cortex led to a significant reduction in tinnitus severity. A systematic review and meta-analysis conducted by Martins, da Silva Souza [23] revealed that tDCS had a moderate effect on reducing tinnitus severity. However, there are also studies that report inconclusive or negative outcomes for tDCS as a treatment for tinnitus. For example, Lefebvre-Demers, Doyon [24] found no significant reduction in tinnitus symptoms following tDCS treatment. The mixed findings from studies investigating tDCS and tinnitus indicate that it is important to design studies to assess the effectiveness of the intervention both at neural and behavioral levels. This will help to provide a more comprehensive understanding of the underlying mechanisms and potential benefits of tDCS as a treatment for tinnitus.

The current study aims to investigate the effects of concurrent high-definition transcranial direct current stimulation (HD-tDCS) and PEI on tinnitus-related negative valence and expand the current knowledge on tinnitus distress treatment. The NfTM proposed that the modulatory effect of anodal-tDCS over the left dlPFC reinforces positive emotion processing, which, in turn, helps with the down-regulation of tinnitus-related negative valence.

Accordingly, we hypothesized that multiple sessions of anodal HD-tDCS over the left dlPFC concurrent with PEI results in the down-regulation of tinnitus negative valence at the neural network level as measured using resting-state functional magnetic resonance imaging (rsfMRI) data.

Secondarily, we hypothesized that induced functional connectivity changes following anodal HD-tDCS concurrent with PEI reduce tinnitus distress as assessed using the Tinnitus Handicap Inventory (THI) [25].

## 2. Methods

### 2.1. Subjects

Patients with constant bilateral subjective chronic tinnitus within the clinical distress stage with a THI score ≥ 18 [26] and not taking medication during the intervention time were included. On the other hand, patients who reported pulsatile or unilateral tinnitus, chronic headaches, Meniere disease, otosclerosis, brain tumors, and current use of medications for depression or anxiety were excluded. Although fifteen patients were initially recruited, only the data from twelve individuals were ultimately analyzed. One patient’s poor image quality and two patients’ incomplete imaging sessions were the reasons for their excluded data.

A total of 12 tinnitus patients (7 females, mean age = 51.25 ± 12.90 years, range 27–67) who had had tinnitus for an average of 9 years (SD = 5.16 years, range 1–17 years) participated in our study (Table 1). This open-label, single-arm pilot for a prospective cohort study was approved by the Ethics Committee for Analysis of Research Projects, Specialized Center of Otorhinolaryngology and Speech Therapy, Hospital das Clínicas da Faculdade de Medicina de Ribeirão Preto, University of São Paulo, Brazil (HCRP no: 55716616.1.1001.5440). All patients gave written informed consent.

### 2.2. Audiological Profile

Before and after each experiment session, a trained audiologist determined the hearing threshold level using pure-tone audiometry (PTA) examination. (For more details, see Supplementary Materials in [27].)

### 2.3. Behavioral Profile

Before each experiment session, patients completed the Portuguese versions of the THI [25] and the 6-item version of the State-Trait Anxiety Inventory (STAI) [28]. For anxiety measurement, we only reported state anxiety scores obtained from STAI, which measures anxiety symptoms in the current moment in contrast to the trait anxiety which measures a generalized predisposition to be anxious.

### 2.4. High-Definition Transcranial Direct Current Stimulation

**Figure 1:**
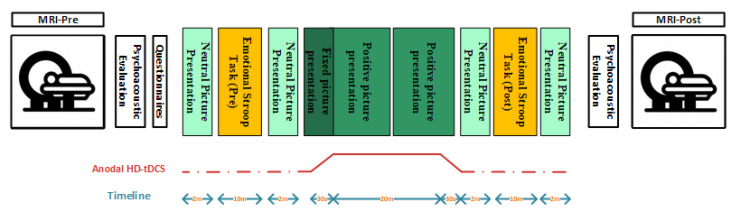
Schematic of protocol.

A battery-driven current source 1 × 1 DC-Stimulator (Soterix Medical, Woodbridge, NJ, USA) and a 4 × 1 distributor (Soterix Medical, Woodbridge, NJ, USA) were administered to deliver 2 mA HD-tDCS for 20 min with a 30 s ramp up and 30 s ramp down. F3 as the recenter electrode was surrounded with four cathode electrodes placed over F1, F5, AF3, and FC3 (Figure 1).

### 2.5. Positive Emotion Induction

We employed a set of validated positive-emotion-eliciting pictures from the Nencki Affective Picture System (NAPS) dataset to induce positive emotion whilst the participants were being simultaneously presented with HD-tDCS over the left dlPFC to reduce the tinnitus negative valence (Figure 1). More details about the HD-tDCS and PEI protocol can be found in our previously published paper [27].

### 2.6. MR Acquisition

Magnetic resonance images were collected using a 3T system (Achieva X-series, Philips Medical Systems, Best, the Netherlands) with a 32-channel head coil. Functional images were acquired using an EPI sequence with the following parameters: 200 volumes, 29 slices in ascending order without gaps, 4 mm slice thickness, voxel size = 3 × 3 mm, field of view = 240 × 240 mm, TR/TE = 2000/30 ms. A silent sequence was used by setting it to the maximum (level 5) “soft-tone” parameter offered by the MRI equipment, which decreases the gradient slew rate, leading to lower coil mechanical vibration levels [29]. Structural images were acquired using a 3D T1-weighted MPRAGE sequence with the following parameters: 3.2/7.0/8 (TE/TR/Flip angle); isotropic voxel of 1.0 mm; field of view (FOV) = 240 (FH) × 240 (AP) × 170 (RL) mm; SENSE = 2. Subjects were instructed to stay alert and remain still. We used cushions between the patient and the head coil to minimize the head movements and earmuffs were also used to attenuate the noise of the scanner. To assure that the MRI scanner noise had not masked the tinnitus sound, we occasionally asked patients to raise their thumb if they were still perceiving tinnitus throughout the scanning. This imaging acquisition was conducted before and after the intervention (Figure 1).

Figure 1: Ten experiment sessions of anodal HD-tDCS over the left dlPFC concurrent with PEI were delivered. Before starting the first experiment session and after finishing the tenth session of the experiment, MRI scans of patients were acquired. Before each experiment session, the patients filled in the questionnaires. Before and after the experiment sessions but not the MR sessions, psychoacoustic parameters of tinnitus were obtained. During the experiment, eighty neutral pictures and two-hundred positive pictures from the NAPS dataset were displayed. The positive emotion induction (positive picture presentation) was concurrent with anodal stimulation over the left dlPFC. The total duration of the experiment was around 45 min.

### 2.7. MR Data Preprocessing

We utilized the MATLAB toolbox CONN v.20.b [30,31] for preprocessing, denoising, and analyzing the fMRI data. The CONN’s default pipeline for preprocessing was used as follows: The functional images were realigned and unwarped, translated by centering to (0,0,0) coordinates, slice time corrected, scrubbed with ART-based identification for outlier scans (intermediate scrubbing settings, with a global-signal Z-value difference threshold of 5 and a subject differential motion threshold of 0.9 mm), coregistered with structural images, and spatially smoothed using a 6 mm Gaussian kernel. Structural images were translated by centering them to (0,0,0) coordinates, segmenting them into GM, WM, and CSF, and normalizing them to the Montreal Neurological Institute (MNI) template. Subsequently, CONN’s default denoising pipeline was performed to remove nuisance variables, including signal within WM and CSF masks, head motion parameters with first-order temporal derivatives, outliers detected during ART, and linear trends. Finally, a temporal band-pass filter (0.008–0.09 Hz) was applied.

### 2.8. Processing/Functional Data Analysis

To examine whether repeated sessions of HD-tDCS over the left dlPFC concurrent with PEI could down-regulate tinnitus negative valence at the neural network level, we selected the dlPFC and amygdala as seeds of interest considering the clinical distress stage of the NfTM [1]. The dlPFC as a key hub in the frontoparietal network takes part in allocating top-down attentional resources [32] toward highly valued stimuli [1]. We further tested more seeds due to their differential activity and/or functional connectivity resulting from tinnitus distress [33,34,35,36]. These included bilateral primary auditory cortices for the auditory network (AN), medial PFC and posterior cingulate cortex for the default mode network (DMN), as well as four seed regions of interest (ROIs) belonging to the dorsal attention network (DAN). The latter seeds were grouped into bilateral posterior intraparietal sulci for DAN1, and bilateral frontal eye fields for DAN2, similar to the study conducted by [36]. Coordinates for the seeds mentioned above were the same as those used in Shahsavarani, Schmidt [35] study. Seeds were generated using the MarsBar toolbox [37] with 6 mm radius spheres centered at the MNI coordinates listed in Table 2.

We performed CONN’s default seed-to-voxel functional connectivity analysis using a weighted general linear model to estimate the bivariate correlation. For each subject, the average time course of the blood-oxygenation-level-dependent (BOLD) signal was extracted from the seed and used as the regressor of interest in the functional connectivity (FC) analysis.

The correlation coefficients between the time series of the seed region and every other voxel across the brain were computed by generating a subject-specific FC map and transformed into a z-score using Fisher’s r-to-z transformation to improve the normality of the correlation coefficients. These Fisher-transformed subject-specific FC maps were then entered into the second-level group analysis using paired sample t-tests to explore rsFC alterations at specific seeds between pre- and post-conditions (specifying post > pre as between-condition contrast). Seeds were tested both unilaterally and bilaterally. For the latter analysis, the connectivity of the two seed regions in each network was averaged together to produce a single representation of the network, similar to the method employed by [36]. The results were significant if they survived at *p* < 0.001 uncorrected thresholds together with a family-wise error (FWE) corrected threshold of *p* < 0.05 at the cluster level, with a cluster extent of 27 voxels. Single-subject Fisher-transformed correlation coefficient values (connectivity values) were extracted from Conn and imported into R-Studio [38] for creating boxplots and scatterplots. Pearson’s correlation analysis was further conducted to test the possible relationship between changes in FC values and THI.

## 3. Results

### 3.1. Effects on Neural Correlates: Resting-State Functional Connectivity

In order to examine rsFC alterations following ten consecutive sessions of HD-tDCS over the left dlPFC concurrent with PEI, we compared the functional connectivity obtained from rsfMRI data acquired before (pre) and after (post) the intervention. We used pre-determined seeds, meant to reflect the connectivity with the bilateral amygdala, FPN, CON, AN, DAN1, DAN2, and DMN. It was revealed that the intervention significantly reduced rsFC between the attention and emotion processing regions at post-intervention when compared to pre-intervention (Table 3).

**Figure 2:**
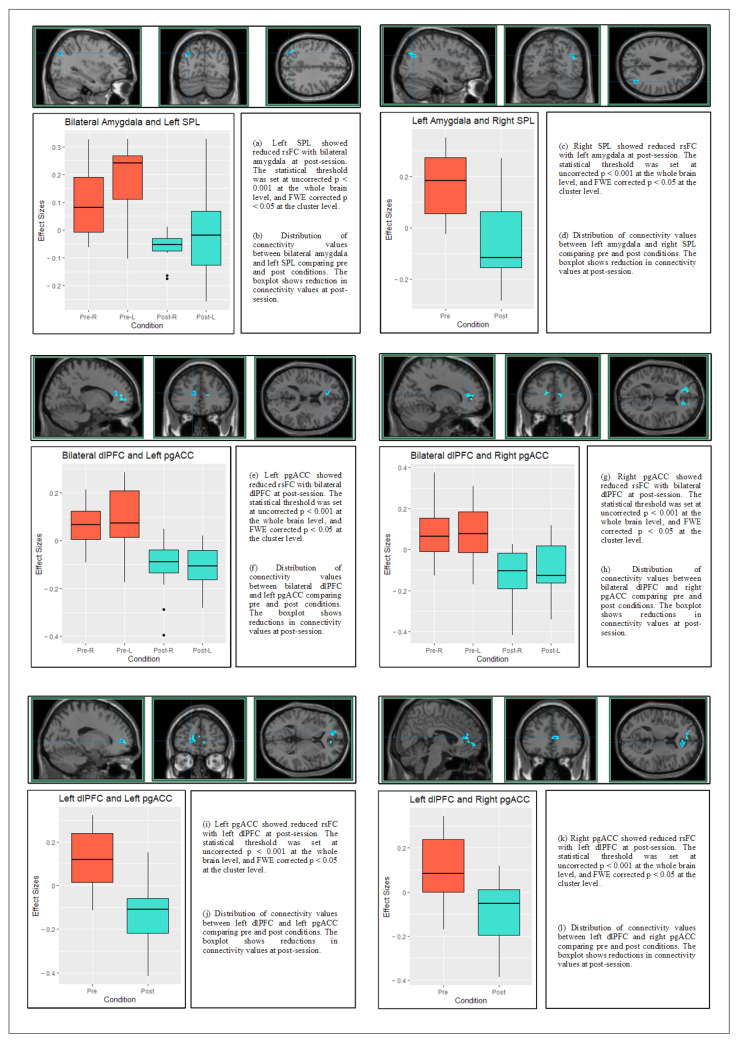
rsFC maps resulting from post > pre contrast and boxplots of the effect size.

Resting-state FC with bilateral amygdala from pre-intervention to post-intervention decreased in a cluster overlapping the posterior part of the left SPL. Unilaterally, the left amygdala showed decreased rsFC with the posterior part of the right SPL at post-intervention when compared with pre-intervention (Table 3 and Figure 2a–d).

Moreover, the between-condition comparison revealed a pattern of reduced rsFC between bilateral dlPFC and bilateral pgACC from the pre- to post-intervention (Table 3 and Figure 2e–h). Unilaterally, the left dlPFC showed decreased rsFC with right superior pgACC and left pgACC at post-intervention when compared with pre-intervention (Table 3 and Figure 2i–l).

There were no changes in the connectivity of other seeds at the established threshold for significance. Although setting the voxel-level significance at uncorrected *p* < 0.01 while maintaining FWE corrected *p* < 0.05 at the cluster level, we observed decreased rsFC between DAN1 and one cluster centered at (MNI coordinate: 26, 6, −14) overlapping with the right-sided putamen, subcallosal area, and fronto-orbital cortex at post-intervention when compared with pre-intervention. The same observation was found using the right posterior intraparietal sulcus as the seed of interest; this time, the suprathreshold voxel was located at (MNI coordinate: 12, 22, −12).

On the other hand, the right primary auditory cortex showed increased rsFC with the post-central gyrus and supplementary motor cortex at post-intervention after reducing the voxel height threshold. With the same liberal voxel height threshold, no clusters emerged for the rest of the seeds.

SPL: superior parietal lobule, pgACC: pregenual anterior cingulate cortex, dlPFC: the dorsolateral prefrontal cortex; statistical threshold was set at *p* < 0.05; FWE corrected for multiple comparisons. The background anatomical image is the single-subject T1 image available at SPM canonical. Anatomical locations were determined using automated anatomical labeling atlas version.3 embedded in xjview toolbox. Blue color represents negative correlations and the center of crosshairs shows the voxel with the peak intensity.

### 3.2. Effects on Behavioral Correlates: Tinnitus Handicap Inventory

**Figure 3:**
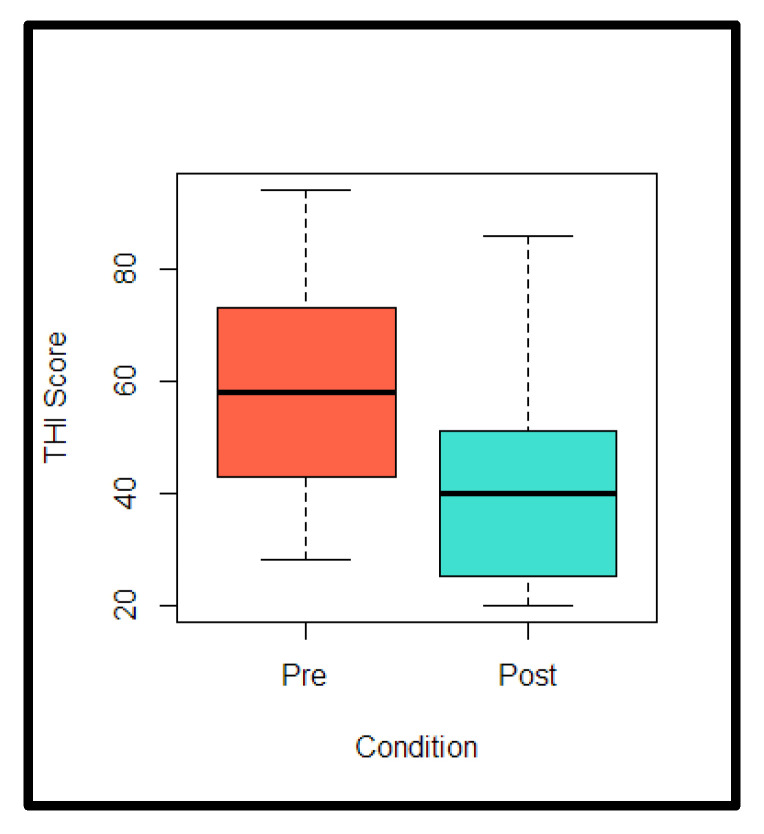
Boxplot for THI scores: pre vs. post.

Paired t-test analysis showed that THI scores were significantly lower at post-intervention compared to pre-intervention [t (11) = 2.77, *p* = 0.0182]. The boxplots in Figure 3 illustrate the distribution of THI scores for the pre- and post-conditions. As Figure 3 shows, a reduction in THI scores at post-intervention is observed when compared with pre-intervention.

### 3.3. Effects on FC-THI Relationship

Pearson’s correlation analysis was conducted to examine whether there was any relationship between changes in FC values and changes in THI scores. The results showed no significant correlation (*p* < 0.05) in the values of both variables neither for pre- nor post-conditions, suggesting that the mechanism behind the FC-THI relationship was not affected by the intervention.

**Figure 4:**
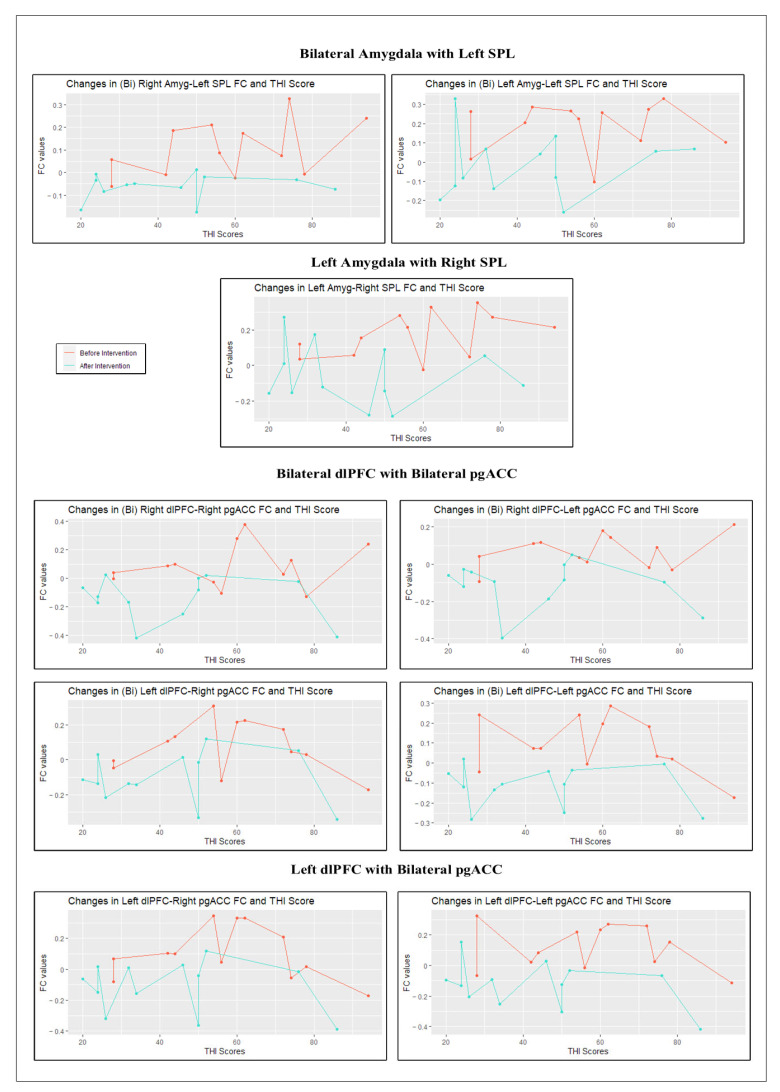
Changes between pre- and post-conditions: FC values in significant regions versus THI scores for each subject.

To visualize the treatment effect, we plotted the variations in FC values against the variations in THI scores between the pre- and post-conditions. As shown in Figure 4**.** and as previously reported, an apparent reduction in specific FC values and THI scores was observed at post-intervention (blue lines) relative to pre-intervention (red lines) Upon visual inspection, one might notice that there is no relationship between the changes in FC values and THI scores neither for pre- nor post-conditions, as was observed after correlation analysis. All in all, the intervention affected the variables independently with no apparent common underlying mechanism (Figure 4).

Figure 4: Points in the plots refer to subject-specific observations for FC values and THI scores before (red) and after (blue) the intervention. Observations belonging to the same condition (pre- or post-) were connected, creating a line to facilitate the comparison. In all of the plots, the placement of the blue points below the red points and roughly under the score of 50, respectively, indicate lower FC values and THI scores obtained post-intervention. Note: for bilateral seeds representing a network, we separately plotted the FC values belonging to the left and the right hemisphere.

## 4. Discussion

When tinnitus is perceived, patients in the clinical stage experience distress because of the corresponding negative valence [1,39]. In light of the NfTM predictions and transcranial electrical stimulation application, we paired the CAAP of tinnitus with the presentation of positively valenced pictures concurrent with anodal HD-tDCS over the left dlPFC aiming at reducing tinnitus negative valence. We delivered the intervention for ten consecutive sessions while the rsfMRI scans and THI were considered as outcome measures at the neural and behavioral levels, respectively.

### 4.1. Neural Correlate

A general picture of the results is a reduction in rsFC between the attention and emotion processing regions, suggesting that the brain is calming down following the intervention.

### 4.2. Amygdala–SPL

The SPL is part of the DAN, which is involved in external attention and goal-directed top-down processing [40,41,42]. The posterior part of the SPL specifically is engaged in selective attention; accordingly, a subset of information is selected for preferential processing [43]. In our study, the decrease in rsFC between the amygdala and SPL after the intervention might suggest that the amygdala assigns less emotional value to the sound accompanied by lowered biased attention.

Taking into account the findings from previous studies, such as increased connectivity between the frontal eye field and parahippocampus [36] suggesting an interaction between the attention and emotion network, enhanced activity [44] in the superior parietal gyrus during resting state, and increased connectivity of the left superior parietal gyrus with various brain regions [45] in tinnitus patients compared with healthy or hearing-loss controls, the observed reduction in rsFC of the SPL and amygdala in our current study may reflect a beneficial effect of the intervention.

Additionally, several studies have shown that anodal tDCS over the left dlPFC can have a beneficial effect on reducing attentional bias. Specifically, studies have reported that a single session of tDCS over the left dlPFC reduced attentional interference in depressed individuals [46] and decreased attention bias to negative content in a stress test [47]. Additionally, tDCS over the left dlPFC diminished amygdala threat reactivity and down-regulated the amygdala in reacting to a threat [48]. However, some studies have not found an effect on attention bias following dlPFC stimulation, which may be due to differences in methodology and study design.

### 4.3. dlPFC-pgACC

The rostral or pregenual area of the ACC (pgACC-BA 24/32), known as the “affective division”, plays a crucial role in the neural circuitry of valuation, and is involved in emotional processing and the assessment of emotional significance in conjunction with the amygdala and other limbic regions [49,50], which are a core part of the neural circuitry of valuation [51,52,53]. Emotional arousal serves as a standard for significance, dictating how brain resources are allocated and boosting sensitivity to environmental cues. Top-down influences, mediated by the frontoparietal and thalamic systems, may lead to increased allocation of cognitive resources and more selective attention when stimuli are seen as emotionally relevant or causing arousal. Frontoparietal attentional systems might receive direct input from regions that specify the motivational importance of stimuli, such as through reciprocal connections with anterior and posterior cingulate cortices, basal forebrain nuclei [54,55,56], or orbitofrontal areas [57,58]. Inputs from these regions might, in turn, affect the response to emotional stimuli [59].

Relevant to our study, reduced functional connectivity between dlPFC and pgACC may indicate that tinnitus is less emotionally significant after intervention, resulting in less attentional resources being allocated to it. The dlPFC may affect distant regions such as pgACC due to anatomical connections [60,61,62]. Previous studies have identified pgACC’s involvement in tinnitus distress via EEG and fMRI studies [20,33,63,64,65]. In one study, tDCS over the right dlPFC led to changes in the resting-state activity in the pregenual ACC, the parahippocampal area, and the right primary auditory cortex, resulting in the transient suppression of tinnitus distress and loudness [20]. In another study, highly distressed tinnitus patients showed greater activity in the pregenual ACC, dlPFC, medial PFC, insula, anterior midcingulate cortex, superior, and middle frontal gyrus, which was positively correlated with tinnitus-related distress [33].

In our study, the center of gravity of the cluster showing reduced rsFC with dlPFC was in the bilateral medial frontal gyrus. Support for the contribution of the medial frontal gyrus in tinnitus distress comes from previous studies [33,34]. In the same study mentioned above, Golm, Schmidt-Samoa [33] reported higher activation in the right medial frontal gyrus among highly distressed tinnitus patients compared to low-distressed ones. This higher activation was positively correlated with tinnitus distress, suggesting that this region is part of the distress network and can be an ideal stimulation site for mitigating tinnitus distress [33]. Accordingly, the reduced engagement of the medial frontal gyrus in our study might indicate a lower level of distress experienced at post-intervention.

The dlPFC has been found to play a role in valence attribution to emotional experiences in various studies wherein anodal tDCS of the left dlPFC has been shown to reduce negative emotional processing in different experiments [66,67]. One study found that 1 mA anodal tDCS over the left dlPFC reduced the perceived intensity of negative emotional valence for negative stimuli but not for positive or neutral stimuli [66]. Another study reported that 2 mA anodal tDCS over the left dlPFC reduced the perception of unpleasantness and personal discomfort in response to images depicting human suffering [67]. More recent studies have provided evidence that the left dlPFC tDCS decreased negative emotional reactivity to aversive content [68,69]. However, the favorable effects of tDCS are sometimes small or difficult to replicate, and this may be due to different stimulation parameters applied across studies [70].

### 4.4. Behavioral Correlate

Comparing the THI scores before and after the intervention, we found that the scores were significantly lowered at post-intervention relative to pre-intervention. This most probably results from the reduction in the engagement of attention and emotion processing regions, reflecting the decreased burden of tinnitus.

Favorable results of dlPFC tDCS on the psychological aspect of tinnitus have been widely reported, although widely varying dose parameters across these studies limit conclusions. For instance, using THI as the primary endpoint, Frank, Schecklmann [71] noted that six thirty-minute sessions of 1.5 mA tDCS (right anode and left cathode) minimally impacted loudness and annoyance [71]. With a similar electrode arrangement and intensity, Vanneste, Plazier [72] carried out a clinical study recruiting 478 patients who suffered from tinnitus and reported that a single twenty-minute tDCS session modulated tinnitus perception among 29.9% of the patients. A significant decline was found in the intensity and distress of these patients when assessed using the visual analog scale (VAS) [72]. On the other hand, in a cross-over sham-controlled study, Faber, Vanneste [73] performed six sessions of anodal tDCS for the left or right dlPFC with a cathode electrode over the contralateral dlPFC. The results of VAS found that both active conditions, regardless of the anodal position, succeeded in decreasing the annoyance associated with tinnitus but not its intensity [73]. However, the above-mentioned studies lack functional targeting and the identification of neural alterations associated with symptom alleviation, which should be taken into account in future studies.

### 4.5. Neurofunctional Tinnitus Model

In agreement with the NfTM, our results corroborated the role of the ECL mechanism in developing tinnitus valence. The NfTM proposed that for tinnitus patients within the clinical distress stage, the CAAP of tinnitus has been repeatedly paired with negative unconditioned stimuli resulting in the generation of tinnitus negative valence. Based on this postulation, in the current study, we used the ECL mechanism to reduce the previously shaped tinnitus negative valence by pairing the CAAP of tinnitus with PEI and HD-tDCS over the left dlPFC. The observed reduction in rsFC with the attention and emotion processing regions and THI scores at post-intervention highlighted the contribution of the ECL mechanism in changing the valence. Such promising results provoke the development of treatments based on the ECL mechanism to reduce the negative valence of tinnitus when paired with positively valenced and high-arousal stimuli such as pictures and films [74]. These stimuli can be presented in a game-like design, app-based format, or via goggles of virtual reality to provide a cost-effective home-based individualized treatment.

Our results both at the neural and the behavioral levels are in accordance with NfTM predictions, i.e., the weaker cognitive–emotional value of the sound lowers the chance of attention allocation and the experienced distress level [1]. Observing the same trend of reduced rsFC between DAN1 and the right subcallosal/OFC at a more liberal threshold adds further support to NfTM predictions. This model, however, did not take into account the involvement of parietal attention-processing regions. Therefore, we propose to explain the differences in attention–emotion interactions between patients with neutral and clinical distress tinnitus while incorporating the parietal attention-processing regions in the model.

Given that the general picture of our results is the interaction between the attention and emotion processing regions, one possible explanation for the respective interaction could be via the framework of the salience network. The salience network is responsible for mediating attention to relevant external stimuli and operating in terms of the associated processing of cognition and emotion [75,76]. However, after the intervention, we did not observe any changes in the rsFC of the anterior insula as one of the main nodes of the salience network. It is suggested that future studies examine whether the anterior insula and dorsal anterior cingulate cortex, representing the salience network, indicate any differential functional coupling in tinnitus patients within the clinical distress stage. A further justification for considering the salience network relates to the correlation between stimulus valence and the salience network [77,78]. If this correlation was verified for tinnitus, the NfTM would need to be revised accordingly.

The NfTM proposes how different brain regions interact, resulting in tinnitus distress. Our observation of alterations in the amygdala, ACC, and lateral PFC at post-intervention confirmed their contribution to tinnitus distress as proposed by the NfTM. More specifically, the finding that the pregenual part of the ACC plays a role in tinnitus distress added further detail to the anatomical structure of the NfTM. Although our findings provided some support for the NfTM, further investigations are still required for model validation.

## 5. Conclusions

The current study aimed to examine whether repeated sessions of HD-tDCS over the left dlPFC concurrent with PEI can down-regulate tinnitus negative valence both at the neural network and behavioral levels. The results indicated attenuated rsFC between the attention and emotion processing regions at post-intervention when compared with pre-intervention. Generally, the brain calms down after receiving the intervention. To illustrate this, we observed that reducing the negative valence of tinnitus could lessen the chance of attention allocation to the sound with a lower level of distress, as was predicted based on the NfTM. However, we still do not know the exact underlying mechanisms which led to the lower rsFC between the attention and emotion processing regions; this might be derived from an improvement in the function of cognitive control regions [79], for which we were unable to find a track. Alternatively, the current findings might stem from the participation of some hub regions mediating cognitive–emotional processes resulting in emotion regulation and controlling behavior [80]. Collectively, future investigations in light of the NfTM are required to better understand the root causes of these beneficial effects.

## 6. Limitations and Future Directions

Varying tinnitus duration and HTL among recruited patients for this exploratory pilot study could impact rsFC [81]. Although we observed preliminary but promising results in the small sample size of this exploratory pilot study, a larger sample size is essential for the confirmatory stage. The absence of a sham-controlled group and the absence of follow-up assessments are among the other drawbacks of our study.

To address the problem of interpretation caused by the widespread modulation of brain activity and connectivity resulting from the stimulation of a given area, future studies should consider using additional active stimulation sites and customized head models for anatomical targeting. Effective connectivity [82] is also strongly advised to improve our understanding of the flow of signals through the regions and networks.

In the current study, the effectiveness of combined HD-tDCS and PEI techniques has been investigated. In future studies, it is recommended to conduct studies on four subgroups, including an HD-tDCS group, PEI group, combined HD-tDCS and PEI group, and also a control group.

## Figures and Tables

**Table 1:** Demographics and clinical and behavioral data of patients before and after the intervention.

	Age (Years)	Tinnitus Duration (Years)	THI		State Anxiety Scores—STAI		LMT dB HL		PTA (dB HL)
							Loudness Pre	Loudness Post	
			1st Session	Last Session	1st Session	Last Session	1st Session	Last Session	
**mean**	51.2	9	57.7	43.3	46.9	39.5	54	47.7	34.5
**SD**	12.9	5	20.1	21.0	15.3	9.8	9.5	12.8	12.7
***p*** **-value**			0.018		0.056		0.049		

THI: Tinnitus Handicap Inventory, STAI: State-Trait Anxiety Inventory, LMT: loudness match test, PTA: pure-tone average, which is an averaged hearing threshold of tested frequencies over both ears.

**Table 2:** MNI coordinates of the seeds used to generate resting-state networks.

Network	Seeds	MNI Coordinates		
		x	y	z
**Emotion** **Processing**	Right amygdala Left amygdala	18 −17	−7 −2	−17 −24
**Fronto-Parietal Network (FPN)**	Right dorsolateral prefrontal cortex Left dorsolateral prefrontal cortex	41 −43	38 33	30 28
**Cingulo-opercular network (CON)**	Right anterior insula Left anterior insula	47 −44	14 13	0 1
**Auditory Network (AN)**	Right primary auditory cortex Left primary auditory cortex	41 −55	−27 −22	6 9
**Dorsal Attention Network 1 (DAN−1)**	Right posterior intraparietal sulcus Left posterior intraparietal sulcus	26 −23	−62 −70	53 46
**Dorsal Attention Network 2 (DAN-2)**	Right frontal eye field Left frontal eye field	27 −25	−11 −11	54 54
**Default Mode Network (DMN)**	Medial prefrontal cortex Posterior cingulate cortex	8 −2	59 −50	19 25

**Table 3:** Regions of significance for post > pre contrast.

Network	Seeds	Region	BA	Cluster Size	Peak MNI Coordinates			Peak Intensity	Cluster-Level p FWE-Corrected	CoG * Regions
					x	y	z			
Emotion Processing	Bilateral Amygdala	L SPL		113	−32	−76	36	−6.80	0.004 *	L Parietal Lobe
	Left Amygdala	R SPL		79	38	−68	28	−6.58	0.038	R Posterior MTG
Fronto-Parietal Network	Bilateral dlPFC	L pgACC	10/32	206	−14	36	16	−7.03	0.00008 **	L MFG-BA 10
		R pgACC	32	107	16	38	12	−5.80	0.0086 *	R MFG-ACC
	Left dlPFC	R Sup. pgACC	32	145	6	36	12	−7.41	0.00084 **	R PreACC-BA32
		L pgACC	10	113	−16	50	4	−6.87	0.0045 *	L Superior MFG

The statistical threshold was set at *p* < 0.05 FWE corrected for multiple comparisons. Anatomical locations were determined using automated anatomical labeling atlas v.3 embedded in xjview (http://www.alivelearn.net/xjview, accessed on 6 April 2021). ** represents a higher significance level. SPL: superior parietal lobule, pgACC: pregenual anterior cingulate cortex, MTG: middle temporal gyrus, MFG: medial frontal gyrus, Sup: superior, * CoG: center of gravity corresponding to each region of significance for post > pre contrast. BA: Brodmann area. L: left, R: right.

## Data Availability

Data will be made available upon request from the corresponding author. The data are not publicly available due to privacy and ethical restrictions.
